# Will we ever teach mathematics again in the way we used to before the pandemic?

**DOI:** 10.1007/s11858-022-01460-5

**Published:** 2023-01-13

**Authors:** Johann Engelbrecht, Marcelo C. Borba, Gabriele Kaiser

**Affiliations:** 1 University of Pretoria, Pretoria, South Africa; 2 UNESP, Rio Claro, Brazil; 3 University of Hamburg, Hamburg, Germany

**Keywords:** Emergency remote teaching, COVID-19, Pandemic, Changing classroom, Online teaching of mathematics

## Abstract

After about two years of emergency remote teaching during the pandemic, the teaching of mathematics is slowly returning to (what used to be called) *normal*. However, after the period of mostly teaching online, there is uncertainty about the extent to which we will return to the way we were teaching before. In this survey paper we attempt to give some background to the impact that emergency remote teaching may have had on teaching mathematics. We examine the possible social implications and then focus on the changing mathematics classroom, focusing on the actual mathematics curriculum, learning design and assessment, the role of collaborative activities and social media, educational videos, and the role of family and parents in future. There are indicators from the literature that educators may not return to the traditional way of teaching entirely, especially in secondary and higher education. We conclude with describing some possible new research areas that have developed through emergency remote teaching, including online education for younger learners, local learning ecosystems, the role of family and parents, instructional design, and the mathematics content of curricula.

*You’re never going to be a lion, and that’s all right, as long as you can act like one when you need to. But right now, as a zebra among a group of lions, when the lions see you they start to think about lunch—and you’re not a guest, you’re on the menu. (*Waldroop & Butler, 2000*, p. 77)*

## Introduction

In a survey paper in 2020, Engelbrecht et al. (2020b) addressed the possible transformation of the mathematics classroom, just as the COVID-19 pandemic struck the world. Since then, the global COVID-19 pandemic has forced this transformation of the mathematics classroom on us—we *had* to change.

Although there is still a strong and growing worldwide debate on whether we are suffering more from the health crisis or from the economic and social crisis that was caused by the worldwide lockdown of economic, social and educational facilities (as discussed by Chan et al., 2021), we do not take a position on this issue as we are facing a new reality. Throughout the world, our lifestyle has changed dramatically and suddenly—we *went online*: we shop online, we meet friends online, and we learn online (Borba, 2021). Whether what we are experiencing economically, socially or in education has been an overreaction caused by the global lockdown or whether it was justified by the intensity of the disease, is not the question that we want to address. In fact, it becomes irrelevant—we have to face the reality that we live in.

Schools and universities were closed in many parts of the world and more than 90% of the world’s registered learners (1.5 billion) were left without education (UNESCO, 2020). Borba (2021) warned of the danger of social inequality that is growing in schools, in that the issue of access is a barrier to some schools and promotes even further social inequality. Being forced to embark on new teaching approaches created an opportunity to understand how human society was responding to challenges in a crisis. Chan et al. (2021) speculated on whether we should document, describe, or explain the crisis or whether we should try to theorise or predict how the crisis is going to play out. We elaborate on the social impact of the pandemic in Sect. 4.

Mathematics education across the world has been widely affected by the pandemic and unprecedented scenarios that required expeditious responses had to be addressed (Chirinda et al., 2021). The agenda of mathematics education was changed. Teachers and students had to make serious changes to the traditional teaching and learning approach, working and learning from home. We rely on digital technology to conduct lessons and other teaching and learning activities and to connect with our students in remote locations off campus. The new reality, in which we live, is now referred to as a ‘new normal’ (Engelbrecht et al., 2020a).

This reality, however, is changing at a rapid pace and what we say today may no longer be valid tomorrow. In normal times, papers on digital technology become dated because the technology changes so rapidly. In the new normal, it is not only the technology that evolves—the external environment that forces us to adapt, is also changing at a rapid pace. Chan et al. (2021) discussed whether the growing worldwide interconnectivity (e.g., through social media) “changes the nature of any potential crisis from a chain of events in a relatively contained ecosystem or society to having global reach” (p. 2). This viral pandemic has given us an opportunity to prepare for similar crises that may happen in future. We do not know—we cannot exclude the possibility of further crises that may hit us, perhaps on an even larger scale.

The COVID-19 pandemic is not the first crisis that we have experienced in mathematics education. Disruptions in education have been happening in many countries, during which times educators have implemented various forms of Emergency Remote Teaching (ERT) and learning through different channels (Chirinda et al., 2021; Hodges et al., 2020). An array of media and technology has been introduced to create hybrid forms of teaching, including e-learning (Ebner et al., 2020), mobile learning (Naciri et al., 2020) and flipped classrooms (Tang et al., 2020). A variety of media platforms have been used to support online teaching and learning, such as learning management systems, instant messaging applications, instant meeting applications, social media and dynamic geometry packages (Engelbrecht & Oates, 2022).

Given that there is little research available on the topic of online education in primary schools, the focus of this paper is on higher education and secondary school contexts and the claims made in this paper relate mainly to these two contexts. Although much of the content of this paper is applicable to mathematics teachers at all levels, in most of the sources that were consulted, the researchers focussed on teaching mathematics at secondary or university level.

A secondary objective with this survey paper—in fact one of the aims of this special issue as a whole—is to relate ideas from literature that seem to articulate some aspects of the mathematics education field from the last two years, that is to give some idea of the rapidly growing amount of literature on the mathematics education situation during and after the pandemic, including the papers in this issue, to which we refer briefly in this survey.

The main aim, however, is to start a debate on how the emergency remote teaching (ERT) strategies that we were forced to introduce during the COVID-19 pandemic, will have a more lasting impact on the way we teach mathematics in future.

So the paper is organised according to the following structure: We begin by discussing emergency remote teaching (ERT) experiments that have been implemented during the pandemic, especially in schools and universities (Sect. 2). This is followed by a discussion on the impact on teachers and the social implications in mathematics education (Sects. 3 and 4). In Sect. 5 we discuss the impact that ERT may have on mathematics education itself, focussing on some theoretical perspectives, the mathematics curriculum itself, instructional and learning design, collaborative activities and social media, educational videos and the role of parents and family. The paper is concluded with a section on new possible research possibilities.

## Emergency remote teaching (ERT)

For years, teachers have been wondering about what mathematics education would be like if the internet were in fact to enter the mathematics classroom. With the pandemic, the discussion about a changing classroom has been accelerated dramatically. The internet, homes and parents have become more important in mathematics education during the pandemic. In this paper we summarise findings and concerns regarding the future of mathematics education, in the post-pandemic world. Learning and teaching in the new normal since March 2020, is now commonly referred to as *emergency remote teaching (ERT)* (Chirinda et al., 2021; Hodges et al., 2020). Hodges et al. (2020) described ERT as

a temporary shift of instructional delivery to an alternate delivery mode due to crisis circumstances. It involves the use of fully remote teaching solutions for instruction or education that would otherwise be delivered face-to-face or as blended or hybrid courses and that will return to that format once the crisis or emergency has abated. (p. 6)

At many institutions, ERT offerings included the usage of learning management systems, procurement of devices for students who did not have access to computers, and providing free data for accessing the course material to ensure that online learning did not become prohibitive to students and staff in terms of affordability (Engelbrecht et al., 2020a).

Worldwide, most teachers had not performed online teaching prior to the pandemic and ERT became quite a challenge as countries began to experiment with ERT to cope with the COVID-19 pandemic. As the expectations have been in the past that we will return to the conventional format, once the crisis or emergency is over (Sulistyani et al., 2021), ERT was seen as a non-permanent shift in teaching that is different from the online teaching and learning that has existed for years (Chirinda et al., 2021; Hodges et al., 2020).

The ERT educational situation is referred to (tongue-in-cheek) as *panic-gogy* (panic + pedagogy) (Kamanetz, 2020). Panic-gogy means addressing the question of how teachers are moving into this environment with their teaching approaches, but it is more than just the didactical approach—it includes understanding students’ practical resources and problems, such as availability of digital devices, internet access, family responsibilities, students sent home who need to find a new place to live, and financial constraints.

We briefly discuss a few examples of studies that have been conducted during the ERT period within the pandemic. We relate teachers’ experiences of ERT at schools and universities, focusing on the ways in which teachers supported mathematics learning, perspectives of students, and choices of resources.

The strategies that South African teachers used to support continued mathematics learning at home during the pandemic, during the phases of moving to ERT and with the gradual reopening of schools later as regulations were relaxed, were explored by Vale and Graven (2022) (in this issue) using activity theory. Their results showed that teachers’ voices can inform possible ways forward for the purpose of managing mathematical learning gaps and meeting on-going learning needs.

The perspective of students was explored in a few studies as well. Thurm et al. (2022) (in this issue) examined students’ experiences with ERT in Belgium, Germany and the Netherlands. In detail, they analysed how these experiences related to their teachers’ beliefs and practices, what didactical approaches and formative assessment practices secondary mathematics students experienced, and which beliefs they held concerning digital mathematics education. They found that even though students preferred regular face-to-face (f-2-f) teaching, they appreciated synchronous delivery of ERT as well.

As for higher education, responses of universities to the COVID-19 pandemic have been reported on in many studies (e.g., Bakker & Wagner, 2020; Hyland & O’Shea, 2021) as most institutions moved to fully online instruction in March 2020. There had been an increase in the use of e-learning in universities over the past decades before the pandemic with a substantial amount of research on online instruction in mathematics courses that has been published (Borba et al., 2016; Hyland & O’Shea, 2021). It is not clear how much of this previous research was incorporated into initiatives during the pandemic.

In a more systemic study from several countries from Southeast Asia, Atweh et al. (2022) (in this issue) described teacher experiences as they dealt with changes in their programme delivery, focussing on the ethical constructs of *responsiveness* and *responsibility* to guide actions in response to a crisis. They identified challenges for mathematics teacher educators to re-design their curriculum, teaching, assessment, and equitable access towards more relevant, productive, and equitable mathematics teacher education. The perspective of equity was taken up by Maass et al. (2022) (in this issue), who analysed the contribution of mathematical modelling to citizenship education, taking the context of COVID-19 as an example, as it has affected the quality of human life of students in the western world.

The perspective of students studying mathematics at university level in Ireland was analysed in a study by Hyland and O’Shea (2021), who investigated the impact of the COVID-19 closures. Their results showed that most students dealt with the rapid changes in a resilient and mature manner, particularly when confronted with adversity. The results confirmed the widespread use of learning management systems such as Moodle and Blackboard to provide access to a variety of resources for students. The asynchronous nature of the recorded lectures and lecture notes uploaded to such learning management systems confirms the notion of “domestication of new media” (Engelbrecht et al., 2020b), a term used to designate the process in which teachers tend just to convert their traditional courses to an online platform.

In a study in South America, Villarreal et al. (2022) (in this issue) analysed the educational experiences of pre-service mathematics teachers enrolled in mathematics teacher education programmes, as the pandemic was unrolling. Their results suggest the need to rethink teacher education programmes, regarding the integration of technologies in mathematics classes, the opportunities offered by hybrid education, and teacher education for distance teaching.

Furthermore, in their study, Hyland and O’Shea (2021) pointed out that lectures, tutorials and support services that had been offered in the pre-COVID times, involving different atmospheres and methods of interaction that students engaged with at various levels, migrated online during the pandemic and largely similar modes of delivery were employed. They considered this issue as important and suggested that tutorials and support services may be priorities in future blended educational approaches (Hyland & O’Shea, 2021). In teacher education, according to Mulenga and Marbán (2020), prospective teachers believe that digital learning will enable a mathematics pedagogical shift to a less traditional way of teaching that is more interesting and entertaining than the customary conventional and traditional way.

Hyland and O’Shea (2021) also examined student perceptions on ERT during the COVID-19 period. They found that students dealt with the rapid changes in a resilient and mature manner, and identified insights from the students’ perspectives that have the potential to improve the teaching and learning of mathematics at university level. Unger and Meiran (2020) reported anxiety and reduced performance among their students brought about by the transition to online learning. Similarly, Adnan and Anwar (2020) described dissatisfaction among students, with the vast majority of their students preferring f-2-f contact with teachers.

In a longitudinal study, Liebendörfer et al. (2022) (in this issue) investigated the issue of how students regulate their learning and specifically their choice of resources and peer learning in university mathematics classes that are fully taught online. The results illustrated the strengths and limitations of digital materials provided by the lecturer and the use of complementary media, also revealing the double-edged role of simple, often anonymous exchanges with few binding forces for either side, and the significance of stable learning partnerships for students’ success.

In a case study by McMurtrie (2021) about a health and humanities programme at the University of Wisconsin at Madison, it was found that 40% of the students did not have internet service that was reliable enough for streaming, and the programme therefore moved to lower-tech, asynchronous learning. Overall, universities realised that it is better to keep it simple—they focus on ‘quick-and-dirty operations’ such as creating discussion boards, posting assignments, or using conference platforms for live or pre-recorded lectures. McMurtrie (2021) also related an essay titled “Please do a bad job of putting your courses online” by Rebecca Barret-Fox in which she attempted to settle people's nerves and make sure they put students first and stuck to the basics. She proposed doing away with the fancy technology and intensive demands on students, saying that it is not realistic to expect the remote-learning transition to be seamless, let alone pleasant. Since she posted her essay about sticking to the basics, she has heard from many colleagues, thanking her for reassuring them that lowering the technology bar, does not imply that they are bad teachers. Her conclusion is that it is not the technology that will save us, but the pedagogy (McMurtrie, 2021).

## Impact on school and university teachers

The imperative on teachers to change their teaching approach radically, had a severe impact on teachers. As it was, teachers were already struggling to balance teaching, research and other obligations, including the work-life balance (Houlden & Veletsianos, 2020; Rapanta et al., 2020). Almost overnight, teachers were asked to become designers and implementers of new pedagogies using tools and resources that few had properly mastered before. Teaching staff on all levels had to prepare and deliver their teaching from home, with all the practical and (especially) technical challenges this entails. Many teachers had never taught an online course (Hodges et al., 2020).

Furthermore, as empirical studies pointed out, many university teachers did not have the necessary pedagogical content knowledge, including technical and administrative aspects of teaching online and pedagogical foundations and knowledge of principles needed to facilitate meaningful online learning experiences (Ching et al., 2018; Rapanta et al., 2020). Teachers and professors had to figure out the intricacies of their learning management systems, unfamiliar conferencing technologies, and new protocols for coursework and tests, as they were trying to find out where students were, physically as well as academically, and what they needed.

In their study Rapanta et al. (2020) focused on the pedagogical preparedness of university teachers with no or little experience in online teaching, as the instructional environment was complex and inexperience brought shortcomings in organisational planning. They pointed out that teachers were inundated with advice, tips and tricks, mostly on tools and resources that they could use, instead of the f-2-f classes. In many instances teachers were lacking the foundational knowledge needed to judge which teaching approach was likely to be successful (Rapanta et al., 2020).

The roles that prospective mathematics teachers assign to technologies for teaching in pandemic conditions were explored in a study in Colombia by Villa-Ochoa et al. (2022) (in this issue). Based on the construct of *Humans-with-Media* and the *Learning by Expanding* theory as frameworks, their study investigated the impact of technology on the Activity System and they discussed the opportunities and limitations of students’ conceptions about teaching and technology during a pandemic.

## Social implications

In this section we briefly discuss social implications of the COVID-19 crisis, which have been explored in several studies. The implications can be grouped into two main categories, social injustice issues that were amplified during the pandemic and personal issues that learners and teachers experienced because of the isolation during the pandemic.

Regarding the issue of equity, Borba (2021) discussed the connections of humans to the virus, how it laid bare social inequality, in that many children across the world just did not have access to internet, and how it changed the agenda of mathematics education. Chan et al. (2021) warned about the dangers of possible inequities that may come with ERT, including unequal access to the internet and to computers and other hardware, as well as the unequal availability of space at home for uninterrupted time for learning. These inequities probably magnify the effects of poverty.

Based on the theoretical framework of *critical mathematics education,* Borba (2021) highlighted that education is not neutral—it can promote equality or inequality. Indicators from Forbes show that social inequality has been growing during this pandemic (Gavioli, 2020). Owners of the big social media and of high tech companies are the winners as people move more and more online: these companies run online social networks and online shopping services, and store digital data in online systems.

Social inequality has been growing in schools during the pandemic, in that as most schools and universities went online and suspended f-2-f classes, the issue of access was becoming a barrier that increased social inequality (Borba, 2021), which led to some extreme cases, where universities even opted to move away from online education because of inequitable access. Educational inequality was high even before the pandemic, and currently it is accelerating at an alarming pace. Vegas and Winthrop (2020) provided alarming statistics on the outcomes of millions of students having been out of school for long periods, and high percentages of children, especially in low-income countries, who failed to master the basic secondary-level skills needed to function in work. It has been the children in the poorest countries who have been left the furthest behind.

On the other hand, for some students in wealthy communities, schooling has been good during the pandemic. Some of them were taught in their homes by a teacher hired by their parents or by their well-educated parents using well advanced internet facilities and modern teaching resources (Vegas & Winthrop, 2020).

The differences in access to remote learning opportunities are not only between rich and poor countries but also within countries. UNICEF estimated that 463 million children—at least one-third of the world’s total, the majority of whom are in the developing world—had no access to remote learning via radio, television, or online content (Vegas & Winthrop, 2020). Moving to online teaching may have had negative implications such as political consequences, in that it could evoke student protests rather than being experienced as pedagogical innovation (Czerniewicz, 2020). On the positive side, some students who had not had access to proper education before, could now be reached, in that good teachers could make their teaching available to a much wider audience.

Engaging a social justice framework to explore the teaching and learning of mathematics during the COVID-19 lockdown in a context of historical disadvantage in South Africa, Chirinda et al. (2021) targeted secondary school mathematics teachers in finding out how teachers responded to ERT during the pandemic. Their findings showed how mathematics teachers became learners themselves, as they had to adapt to digital teaching, find solutions to unfamiliar problems, and acquire knowledge from a larger mathematics education community around the globe.

Teachers with low salaries were not likely to have the best mobile phones, laptops, or internet access, and differences between the ‘haves’ and the ‘have-nots’ were amplified by COVID-19. Overall, the role of mathematics education for resisting inequality in the world, is a topic that should be researched (Borba, 2021).

The second concern about the social impact of the pandemic has more to do with the students’ and teachers’ personal experiences. The increased isolation caused a lack of motivation during the closure, and students were more likely to experience increased anxiety during the pandemic (Huang & Zhao, 2020). Hyland and O’Shea (2021) recommended that educators should be extra vigilant of students’ well-being, given the reduction in access to general support services such as counselling. Unger and Meiran (2020) called for students’ mental health to be monitored during pandemics.

The issue of priorities was raised by Chan et al. (2021) as in crisis time we tend to focus rather on more important issues, such as the well-being of our families, our communities and our students. As teachers, we may consider it important to help our students achieve their immediate needs, even if those needs relate to systems that we do not necessarily agree with. Or we may prefer addressing the bigger problems, attempting to change systems.

Packer (2022) was concerned about how the public-school system in the USA will survive the pandemic. Teachers, whose status had been in decline for a long time, were leaving the field. In 2021 nearly one million people resigned from public education in the USA, 40% more than in the previous year. Students were leaving the public system as well. Since 2020, nearly 1.5 million children were taken from public schools to attend private schools or to be home-schooled (Packer, 2022).

Already in 2005, Borba and Villarreal (2005) synthesised how the notion of humans-with-media could be understood, based on the work of Lave (1988) and Levy (1993), contributing to the notion that learning is social, not only in the sense that it involves more than one person, but that it also involves ‘things’, e.g., pieces of software, hardware, and the internet. Consequently Borba (2021) showed that different media shape humans, but also gave evidence of how humans shape technology. He reported on the interaction between a piece of software and how high school students would interact with the software and with the teacher.

## The changing classroom—impact on mathematics education

In a review of research on online learning in mathematics, Engelbrecht et al. (2020b) investigated how mathematics classrooms have developed with the growth of the internet and interactive devices. Although some people claimed that f-2-f interaction is fundamental to any learning that occurs in mathematics education, everyone was aware of the fact that classrooms are changing. We could speak of a *classroom in movement* or a *distributed classroom—*it moves from the traditional cubic space to a combination of a classroom with a bedroom for one student, an office for another, and some kind of computer centre for others (Borba, 2021).

Morin (2016) described how students exposed to online learning have their own concerns, new motivations and new challenges with respect to education. Hyland and O’Shea (2021) claimed that massive open online courses, blended learning, flipped classrooms and other technological approaches used before COVID-19, might not be as fully used in current post-COVID-19 teaching as one might have hoped. They considered these approaches to be noticeably different from current instruction in which many teachers just use traditional versions of courses as models for the online versions without taking full advantage of the new opportunities afforded by the technology—in fact, many teachers just published their lectures online.

More generally, Engelbrecht et al. (2020b) posed the question of whether digital technologies would be able to provide alternative ways to support mathematics education. Based on the theoretical construct of humans-with-media, Borba (2021) connected the pandemic to three different trends in mathematics education, namely, the use of digital technology, philosophy of mathematics education, and critical mathematics education. He discussed the possibilities and drawbacks of having more and more online education using the notion of humans-with-media and its perspective of collective knowledge production involving human and non-human actors.

In a systematic literature review on the use of flipped classrooms, Cevikbas and Kaiser (2022) (in this issue) focused on opportunities and pitfalls regarding pandemic-related issues. Their results demonstrated that flipped classrooms can be a promising pedagogy that has numerous benefits for mathematics teaching and learning, although it cannot be seen as panacea for pandemic-related issues, as it also has several significant pitfalls. Their review contributed to gaining insight into successful implementations of flipped classroom pedagogy, not only during the pandemic but also beyond the pandemic.

In the following, the impact of the pandemic on changing the mathematics classroom is discussed relating to possible future constructs and theoretical perspectives to analyse the changing classroom, the mathematics curriculum, instruction and learning design and assessment, collaborative activities and social media, and the role of parents and family.

### Possible future constructs and theoretical perspectives

A number of theoretical frameworks exist (Brown, 2017) for using digital technologies in mathematics education, e.g., *Instrumental Orchestration* (Trouche, 2004); the *Structuring Features of Classroom Practice framework* (Ruthven, 2014); *Pedagogical Technology Knowledge* (Thomas & Hong, 2013); and *Enactivism* (Khirwadkar et al., 2020). In instrumental orchestration, digital tools are shifted from a tool to an instrument—the focus is on how the teacher supports this process. The framework by Ruthven (2014) focuses strongly on how this occurs and includes structuring features of classroom practices that influence how digital technology is integrated (Brown, 2017; Ruthven, 2014). Thomas and Hong (2013) introduced the construct pedagogical technology knowledge (PTK), which includes the teachers’ perspective on the technology, their familiarity with the technology as a teaching tool, and their understanding of mathematics. It has been assumed that teachers with high levels of PTK would focus on mathematical concepts, appreciate the mathematical benefits of using technology and take a multi-representational approach, whereas teachers with a low level of PTK would focus on operational matters, procedures and technical skills (Brown, 2017). Before the pandemic, teachers were using technology either to a limited or to a more sophisticated extent. Those with a low PTK, used it to a lower extent and focussed on operations, procedural and technical aspects of technology use. Others, with a higher PTK, used technology with a stronger multi-representational approach.

Callaghan et al. (2022) (in this issue) used an *enactivist* approach, including the inputs of all role players (teachers, learners, policy makers and the community) to address the situation that has evolved. In an enactivist perspective, cognition grows from a network of interactions among agents and their environment. Since existing prescriptions on how to develop a mathematics environment limit the viability, in an enactivist approach the point of departure is that the mathematics community does not simply react to the pandemic as a problem—it is seen as an opportunity for the mathematics community along with all role players to collaborate and to adapt and redesign mathematics education within the constraints related to the pandemic (Khirwadkar et al., 2020). Hoyles (2018) agreed that mathematics teachers must be part of the transformative process as co-designers to transform mathematical practice using digital technologies. As already suggested in this paper, the perspective anchored in the construct humans-with-media is evolving and emphasises the role of non-humans in mathematics education: Besides digital technologies (e.g., Geogebra, internet, online environments), homes, and other types of technologies emerged as having agency in mathematics education.

So, in re-imagining the way we teach mathematics, an enactivist approach could facilitate the involvement of the entire relevant community, interacting with all agents and their environment—to move from the pre-pandemic pedagogy and the panic-gogy phases during ERT, to a post-pandemic pedagogy (Callaghan et al., 2022).

### The mathematics curriculum

The COVID-19 pandemic made the public aware of the important role that mathematics plays in our society. The mathematical topics involved in modelling the course of the pandemic mathematically inspired the imagination of a much wider section of the general public, and students were interested in learning more about these topics. The question of curriculum is widely discussed in this special issue of *ZDM – Mathematics Education*. The mathematics that has been getting publicity during the pandemic was discussed in a number of papers in this issue. These would include, e.g., exponential growth, interpretation of graphs, and other mathematics content used by the media to interpret the pandemic.

A very old, but still crucial question has been becoming particularly popular in this crisis time: *What mathematics should be taught when?* This question needs on-going thought, discussion and research and there will never be a final answer. The answers change with time. New realities, such as the pandemic, may give new insights concerning what is currently relevant.

Mathematics educators and curriculum developers need to identify mathematics that is needed for modelling and interpreting crises so that this mathematics can inform the public. This could be translated to the following question: What mathematics should every citizen know? Chan et al. (2021) broke this issue down to asking what mathematics everyone should know to understand the following four current issues of relevance, but they may change and evolve in time:

- interconnectivity,
- climate,
- biodiversity,
- wealth distribution.

When we develop new curricula in mathematics education, we should include these relevant topics to inform the general public about the mathematics—citizens should be equipped to understand the mathematics they will experience in the world (Chan et al., 2021). Recently, a substantial amount of material has been published by mathematics educators on relevant mathematical topics that could be included in school and university curricula to address this need. In their study (in this issue) Meyer and Lima (2022) emphasised the importance of using mathematical models and mathematical modelling, using difference equations and ordinary differential equations to model scenarios of the pandemic and the general dynamics of infectious diseases, making provision for possible vaccination, supporting Borba’s (2021) suggestion that exponential functions should be taught from early ages to university, which may enhance understanding of sigmoids and curves related to the pandemic.

There is also quite a strong focus on developing a public understanding of mathematics that is used by the media to explain and predict trends in the pandemic and other global phenomena. In their study Siller et al. (2022) addressed the way the media present data on the pandemic, referring to growth models, which attempt to explain or predict the effectiveness of interventions and developments, as well as the reproductive factor. Since these reports sometimes appeared contradictory or even false to students, they showed that basic mental models about exponential growth are important for understanding media reports of COVID-19. They also highlighted how the COVID-19 pandemic can be used as a context in mathematics classrooms to help students understand that they can and should question media reports on their own, using their mathematical knowledge. Lim et al. (2022) (in this issue) showed how the proliferation of data visualisations about the pandemic, particularly those found within the media, have brought a corresponding growth of new data visualisation forms. Building on current efforts to expand the teaching and learning of data practices in mathematics education, they provided examples of innovative data visualisations and examined their pedagogical affordances and constraints in eliciting students’ emotions and bodies as a generative direction for making sense of COVID-19 within a mathematics or statistics classroom. Cantoral et al. (2022) (in this issue) investigated the ways in which the press used graphs to provide information about the pandemic, in order to provide theoretical references to teach mathematics that allow students to understand the information provided in these types of graphs. They concluded that there is a need to incorporate in the teaching of mathematics, a conceptualisation of exponential growth that is linked to the use of graphs in the press.

Kwon et al. (2021) investigated the use of graphs in Korea’s news media during the COVID-19 outbreak, looking at implications for future teaching and learning of graph literacy in mathematics courses. After examining the Israeli public’s understanding of the mathematics that is required for understanding the pandemic and predicting its spread, Heyd-Metzuyanim et al. (2021) demonstrated that adults' engagement with such information may be limited.

### Instructional and learning design and assessment

RAPANTA et al. (2020) characterised instructional and learning design as a process that teachers use to plan, implement, and evaluate their instruction. A good design consists of clear learning objectives, carefully structured content, relevant student activities, controlled workloads for teachers and students, integrated external resources, and assessment connected to the learning objectives. Careful design of learning activities, enabling students to reach the learning outcomes, has been considered as the “the essence of an online course” (Carr-Chellman & Duchastel, 2000, p. 233). The different design elements, synchronous, asynchronous, online, offline, should be carefully balanced, regarding clear communication, an appropriate adequate level of difficulty for students’ capabilities and expectations, being related to contexts to increase students’ engagement, and being accessible to all—taking the available infrastructure into account (Rapanta et al., 2020).

It is clear that the transition to the new pedagogy shifted the focus on to students as responsible for their own learning. This is even more current for assessment, which is growing strongly into a continuous activity with the cognitive expectation of self-regulation (Rapanta et al., 2020). Hyland and O’Shea (2021) reported that students prefer a combination of continuous assessment and online examinations. They reported that many students prefer live synchronous (f-2-f or virtual) lectures and tutorials and consider the opportunities for interaction with teaching staff and other students as important. Meehan and Howard (2020) supported this view in their study and claimed that students feel that the sudden shift online adversely affected interactive activities.

In the post-pandemic era, the opportunities and affordances that we were exposed to during ERT provided by well-designed online learning environments and their capacity to be stimulating, inclusive and flexible, should still be included in student-centred programmes in higher education. Personalisation of learning and flexible pathways can only be provided with a good course design that takes into consideration the full context of the teachers’ and learners’ humans-with media experiences. If teachers invest time in developing learning activities that address learners’ social and cognitive needs, learning outcomes will improve (Rapanta et al., 2020).

Online assessment has long been investigated and implemented (Sandene et al., 2005; Sangwin, 2012), and likewise there have been a growing number of studies that examined peer interactions in the online space (e.g., Goos & Geiger, 2012; Larkin & Jamieson-Proctor, 2015; Mojica-Casey et al., 2014). The need for the development of authentic formative assessment activities that can be used online asynchronously and which facilitate active student learning and peer collaboration, was pointed out by Engelbrecht and Oates (2022). Responding to the challenges of the COVID-19 pandemic, we need to design new learning activities that combine the three types of an online presence, namely, social, cognitive and facilitatory.

Sandene et al. (2005) posed some key issues for technology-based online assessment in mathematics focussing on measurement implications, including equity, efficiency compared with paper and pencil, and operational implications. Sangwin (2012) described the development of STACK, an advanced general computer assessment system for mathematics, with an emphasis on formative assessment, which allowed students to enter mathematical solutions in the form of algebraic expressions, as opposed to the more common use of automatically generated multiple-choice questions.

Cusi et al. (2022) (in this issue) focussed on developing assessment schemes that maintain the pedagogical continuity that educational institutions typically require, during the lockdowns and even in the post-lockdown emergency period. They investigated teachers’ perspectives on the assessment difficulties with which they had to contend, the techniques adopted to deal with these difficulties, and the ways in which the lockdown experience could affect the future evolution of teachers’ assessment practices.

Domingues and Borba (2021) discussed mathematics video production by students, as a facet of the transforming classroom. They noticed that students develop further mathematical knowledge when they express this knowledge in a video, which enables a new language with less rigour but with a clearer objective, as it gets students’ attention while emotional and aesthetical concerns are considered. They found that, for teachers and students, producing a mathematics video is more than ‘just turning in a list of mathematics exercises’, and they are convinced that producing videos is a fruitful tool for the teaching and learning of mathematics. They presented a survey of the emergence of videos in mathematics education, showing how the changes in mathematics education during the pandemic involved this form of expressing mathematics and mathematics education.

Although mathematicians had interacted frequently through videos, this newly established trend gained momentum during the pandemic and currently, on occasion, some students use video-classes even more often than books. In countries such as Brazil, students and teachers are also using videos in a different form: teachers are fostering students to produce videos themselves that show what kind of mathematics students have learnt, including videos that contain didactical material. Inspired by the previous work of Canadian authors (see Gadanidis & Scucuglia, 2020, for a review of part of this work), digital videos in mathematics education seem to have started a new trend.

As reported by Domingues and Borba (2021) the generation of videos and its connection to mathematics video festivals contributed to the transformation of the traditional classroom, and were also important during the pandemic. They described an example of a mathematics festival consisting of an online environment (www.festivalvideomat.com) and a set of f-2-f annual events. In 2020, these festivals were organised virtually as well. Currently, there are about 600 mathematics videos on the website. The website contains details about news, dates, announcements, guidelines for submitting assignments, and videos that have been submitted. This environment is a locus of research, and a space for exchange and discussion of mathematics ideas between students, teachers and the whole community outside the school.

The combination of research, teaching and outreach work seems to add importance at this time when parents and other role players, such as family members or neighbours, become increasingly important for education, as discussed in the next section of this paper.

### Collaborative activities and social media

Since Vygotsky’s (1978) work, collaborative learning activities have been employed at an increasing rate all over the world. Today’s employers seem to view the capacity to collaborate in solving problems as similar or even more important for tomorrow’s workers than content knowledge—they are looking for people who can work effectively in teams (Jackson, 2013). Teachers are trying to build students’ collaborative skills, but developing these skills has always proved somewhat challenging. In traditional classrooms in school and university where a teacher stands at the front of rows of students sitting at desks, collaboration was often difficult; modern teaching approached have relied on multiple strategies to encourage collaboration, including using technology to promote team projects, linking their students to classrooms across the globe (Jackson, 2013).

A growing number of research studies focussing on technology-supported collaborative learning, have appeared. For example, the paradigm of *computer-supported collaborative learning* as a dynamic, interdisciplinary field of research, already developed nearly two decades ago (Resta & Laferrière, 2007), focused on ways in which technology can facilitate the sharing and creation of knowledge and expertise through peer interaction and group learning processes. It included using technology to support synchronous and asynchronous communication between on-campus students as well as students who are geographically distributed.

As in any mathematics course in school and in university, knowledge construction is acquired not only by accessing the information, but also by the interaction that occurs among students and teachers; interactions need to occur collaboratively among participants (Engelbrecht & Oates, 2022). Therefore, the focus in education has been changing from a situation of students passively absorbing information from an educator who is teaching by writing on the blackboard—*pushing* knowledge—to a stronger student driven approach, where students take control of the learning process—a *pull* process (Bassendowski & Petrucka, 2013).

An environment that supports the development of communities and collaborative discussion opportunities can assist students in comprehending and synthesising information, as independent and critical thinkers (Engelbrecht & Oates, 2022). Social media have been used as a significant tool in providing such an environment, which supports self-directed and self-determined learning (Blaschke, 2019; Schuetz, 2014) and foster a sense of belonging, facilitated by collaboration and communication (Balakrishnan et al., 2017; Goodyear et al., 2014).

In fact, social media, with their key social networking capabilities, seem to be an ideal way of facilitating collaboration, and many studies referenced both the growing use and importance of social media in mathematics education, already before the pandemic (Selwyn & Stirling, 2016). For example, Ng and Latif (2011) described how Facebook and blogs could be used in a blended university mathematics course to complement the f-2-f tutorials in order to foster communication and knowledge exchange among learners, peers and tutors in a more informal manner.

An examination of social media sites on the internet provided some insight into how students use social media. Comment social media streams^1^ (which at times can be surprisingly deep) form an important component in the social interaction leading to learning (Engelbrecht & Oates, 2022).

However, while many such studies did indeed support and extol the benefits of social media, others explored the more nuanced impact and issues with respect to academic and cognitive factors, and potential limitations (Staines & Lauchs, 2013; van Bommel & Liljekvist, 2015). So it is clear that much research is still needed on the role of social media in mathematics education (Mulenga & Marbán, 2020; van Bommel & Liljekvist, 2015).

The dynamics of social media that capture the imagination of our students and teachers is also changing rapidly, and sometimes differently for students and teachers. Where *Facebook* was on top previously, most younger people now use *Instagram* and *Whatsapp,* and *TikTok* is becoming increasingly popular.

Engelbrecht and Oates (2022) addressed three essential questions concerning the role of social media and we do not dwell on these here: these question are still open and need to be researched, especially due to their relevance for changing mathematics education in pandemic times:

- Has anything changed? How does the use of contemporary social media differ from previous use?
- How might social media best be used and what pitfalls do they entail? How can we combine social media tools with good teaching practices to contribute effectively to student engagement?
- How valuable are social media for learning mathematics? What evidence of learning is there in comments in blogs and comment streams on social media platforms?

### Role of parents and family

With many children staying home and working online, the format and boundaries of the traditional classroom were changed and suddenly parents and family members had to engage on tasks that formerly were the responsibility of the teacher. Parents and caregivers had to become full-time educators, trying to balance the competing demands of child rearing, schooling, and their own employment (Biag et al., 2021).

Already for decades a strong relationship could be identified between parental involvement in the education of their children, and children’s performance. For example Taylor (2020) found that an educationally supportive home environment, including intellectually engaging materials in the home, such as books, newspapers and educational toys had a positive impact on learning. Even when less educated parents became involved in their children’s education, children were less likely to experience learning disadvantages (Dearing et al., 2006).

During the pandemic many parents were not equipped to assist their children with their studies and many were just too busy with their own programmes to find time to work with their children. Even parents who were able to assist, had to find a balance between supporting their children and applying excessive pressure. There is also the issue of the parents and the teacher presenting the subject matter differently, causing confusion with learners (Hill & Tyson, 2009).

A positive relation was found by Taylor (2020) between learner performance and parental practices such as reading to their children, checking their school bags, keeping track of their reading levels and assuming some degree of responsibility for their children’s reading.

## New research possibilities

With the forced exposure to ERT during the pandemic, many questions and uncertainties emerged; we clearly need proper research to learn more on what works and what does not. A very real immediate problem is how we communicate our research findings. Since 2020, academic conferences had to be conducted in online or hybrid format. Engelbrecht et al. (2022) (in this issue) addressed the issue of the nature and *format of academic conferences* in mathematics education. Surveying active research academics in mathematics education, they investigated the future impact that a change to virtual or hybrid conferences may have on the format and nature of mathematics education conferences. Their findings indicated that although academics are pro-actively thinking about alternative conference formats, the proven value of f-2-f conferences is still very real, showing that it may be too early to have a clear vision of the future format of academic conferences.

It has been noted that there is little research on *online education associated with levels below high school.* With many educational systems forced to go online because of the pandemic crisis, the argument to use technology is very strong. It is likely that we will have more research associated with this new reality. As this theme develops, mathematics education will have to deal with structural issues, such as the participation of parents or responsible other parties in education as well as the role of homes (Borba, 2021).

*Educational innovation* has been moved suddenly from the margins to the core of the education system, providing opportunities to identify new strategies that can develop young people and prepare them for the changing times, such as the smart use of technology and data that allow for real-time adaptation (Vegas & Winthrop, 2020).

The idea of *local learning ecosystems* was suggested by Vegas and Winthrop (2020). There is wide evidence that young people engaging in diverse learning opportunities outside of school—from classic extracurricular activities such as music lessons to non-formal education programming—can be quite helpful in boosting the skills and academic competencies of marginalised children. Examples of emerging models include the *Educacio360*^2^ initiative in Spain and the *Remake Learning*^3^ initiative in Pennsylvania, USA, where school districts have engaged to offer general learning opportunities to families and children. Using the experiences registered during COVID-19 will hopefully harness new energies and relations between schools and communities in working together to support children’s learning.

As mentioned before, the pandemic has introduced new actors in the community to support children’s learning. The pandemic has involved community sections that traditionally were not actively involved in education. Relationships and partnerships between teachers and parents developed in ways that we never thought of before. Schools formed new relationships with social welfare organisations, media companies worked with education leaders, technology companies partnered with governments, and local non-profit organisations and businesses contributed to supporting children’s learning in innovative ways (Vegas & Winthrop, 2020).

The importance of the actual curriculum is becoming blurred as students increasingly turn to informal educational platforms and social media for their learning interactions. Many students of today and tomorrow want to be involved not only in in how they are taught but also in what they are taught (Dekker, 2021; Mkandawire et al., 2018). Not unlike an infant learning to speak, they want to decide on what mathematics they learn and how, in a pull approach, rather than a curriculum that is pushed on to them by the educational system (Engelbrecht & Oates, 2022). So the *role of external resources*, such as social media (but some that may be traditional) should be an interesting topic for research in the near future and may become a relevant research topic in mathematics education.

Regarding *digital collaboration and social media*, Engelbrecht and Oates (2022) identified a number of issues that seriously need in depth research and investigation.

- What technology should students use to support their own mathematical learning as well as collaboratively the learning for other students?
- How can social media tools be combined with the best practices in teaching and contribute effectively to student engagement and the development of deeper mathematical understanding?
- How can we better understand the critical processes or mediating variables that are needed, such as structuring of tasks, ill-defined problems, student engagement, teacher scaffolding, and the ways they combine to create online written discourse in meaningful mathematical settings (Resta & Laferrière, 2007)?
- How should we design assessment in mathematics education to encourage online collaboration and provide students with formative feedback?

## Conclusions

One positive result of the pandemic is that there is newly found public recognition of how essential teachers and schools are in society and an opportunity to leverage this support for greater realisation of the importance of education and the role of teachers in society. Currently there is a much greater public appreciation of the importance of the role of teachers. As parents struggled to work with their children at home due to school closures, public recognition of the essential caretaking role schools and teachers play in society has increased significantly (Vegas & Winthrop, 2020).

March 2020 will be remembered as the time all the world’s schools closed their doors. As teachers around the world struggled with little forewarning to enter into ERT, parents and families around the world, had to face the shock of *life without school.* Before the pandemic, the public sector had come to rely on schools as a given—an anchor around which they organised their daily programmes (Vegas & Winthrop, 2020).

Suddenly politicians and other prominent people in society urged that education be prioritised. This broad recognition and support for the essential role of education in daily life can be found in media reports across the world. The global education community inspired the UNESCO based broad consortium, with the newly formed *Save our Future* campaign, bringing together a wide group of role players, to advocate for sustained education funding. Unexpectedly, vast numbers of parents and families around the world share the long-standing concerns of the most vulnerable families: they need a safe and good enough school to send their children to (Vegas & Winthrop, 2020). This could be the moment in history when the important role of education in the social and economic stability and prosperity of society becomes more obvious and better understood by the general population. It is an ideal moment in time to deliberate on a vision for how education can emerge stronger from the global pandemic than before and how we can propose a path for capitalising on education’s newfound support in virtually every community across the globe.

For many years, educationists have been pleading to raise the status of teachers in society. “Use the top people of today to teach the next generation” is a quote that has been used often in this regard, without much impact: in many countries *becoming a teacher* is not the most popular option for high school students, at least in many western countries. The reality, amplified by the pandemic, has brought the issue of education into the living rooms of poor, middle class and elite parents around the world. And, at least for a moment, these parents are getting involved in supporting education. We should exploit this opportunity to make sure that we get the best people of our generation in the teaching profession.

Returning to the question asked in the title of this paper, namely, whether we will ever teach mathematics in the same way we used to, the answer is an easy *no never—*at least for most people in higher and secondary education. We are fully aware of the fact that institutional systems are slow in changing, but all indications are that the way we teach is changing. We are also aware of some (especially under resourced) primary schools where teaching of mathematics has tended to revert to pre-pandemic f-2-f format, but for the higher education sector and for teachers and students in secondary schools, where basic resources for internet connectivity and usage are available, the answer is an emphatic *no*.

In being forced to teach differently, we learnt much. Too many of the actions that we were forced to embark on, worked so well that we do not want to stop doing them.

Granted (and as we have mentioned), many of our students are happy that we are now returning to f-2-f lectures in the second half of 2022—they are looking forward to physically seeing and experiencing their fellow students and their teachers. The extent to which we will return, however, does not entail that we will ‘carry on as usual’. Many universities will now move to ‘hybrid’ teaching, where along with the f-2-f lectures there will be strong online activity.

In fact, opinions concerning the extent to which we will return to the conventional approach vary significantly and this issue will have to be ironed out. With this paper we hope to initiate (or at least contribute to) a dialogue on what could be achieved in the medium to long term.

Vegas and Winthrop (2020) recommended five actions, which need to be focussed on after the pandemic. These actions include (amongst others) a focus on the instructional core, deploying education technology and forging stronger, trusting relationships between parents and teachers.

It is essential that when we return to (an either ‘old’ or ‘new’) normal, we implement what we have learnt during the pandemic. So far we have had little time to reflect on what we were forced to do in a very short time. Many emergent decisions had to be taken at institutional and personal level, and many practices were changed urgently. Little time, however, was devoted to, what Schön (1983) calls *reflection-on-action*. We need to now spend time for reflection-on-action (Rapanta et al., 2020) and we hope that this paper will contribute to this process.

The outcomes of such a reflection can include greater clarity on course design, the role of the teacher and assessment in online learning. So we would like to see the current pandemic

as a catalyst that highlighted the need for educational change towards more flexible models and practices that best respond to the complexity and unpredictability of today’s fast and interconnected but and still fragile society. (Rapanta et al., 2020)

We consider that the educational experiences that we had during the pandemic can serve as catalysts for teachers to experiment with new ideas, explore creative alternatives and reflect on their own practices (McKenney et al., 2015; Rapanta et al., 2020). Studies included reports about mathematics teachers who experienced the pandemic as a prompt to re-examine their teaching (Albano et al., 2021; Gosztonyi, 2021; Maciejewski, 2021). We as academics in mathematics education must be part of the process of reflection and planning, and universities, now more than ever, should invest in professional development of university and school teachers, for the purpose of helping them to be updated on effective pedagogical methods with or without the use of online technologies (Rapanta et al., 2020).

Our experiences with the pandemic should inspire our confidence that mathematics and good mathematics teaching can make important contributions towards resolving the problems and issues that came with the pandemic, and we should make sure that we learn from this experience in order to improve our teaching.

Many of our students are Gen Z students (born between 1997 and 2012), as described by Engelbrecht et al. (2020b). To them, interactivity and communication are of great importance. Hyland and O’Shea (2021) found that students’ ability to collaborate was impacted by problems with peer communication and they reported as follows:

It is interesting to note that although it is likely that many of these students are part of the generation that uses social media the most, they still value the face-to-face nature of communication in lectures, tutorials and support centres. (p. 18)

As raised by Chan et al. (2021), the central question for both mathematics teaching practices and for research approaches, is, *What should be changed and what should be maintained?* A possible way to move toward this decision is following an enactive approach consulting with a wide range of stakeholders, including students, teachers, parents, policy makers, and other people from a range of contexts.

In 2020, Engelbrecht et al. (2020a), in an editorial in this same journal, asked the question, *Will 2020 be remembered as the year in which education was changed?* It is probably too early to tell yet. In fact, education is always evolving, always changing. But at this early stage it certainly seems that the changes in mathematics education that we experienced with the pandemic will be seen as a leap in the growth curve.

Let us conclude and return to the quote at the beginning of this paper. We need to realise that the entire education scene, including (or particularly) mathematics education, is changing rapidly. After COVID-19, we all have understood that there is a need to take stock now, to consider what we have learnt about mathematics education during the crisis, and to realise that all of us have to be part of the solution. Many of our students complained about the absence of f-2-f lectures and meetings and looked forward to f-2-f classes resuming. Other students enjoyed the online environment and did not want to return to a f-2-f environment. We need to decide to what extent we will return to the previous approach—what elements should come back and what elements of ERT should be retained and developed.

As this quote implies: *If you are not at the table, you could be on the menu*. If we (as zebras) do not act as lions and have our voices heard by those who make decisions, others will decide about the future of mathematics education. If these *others* (the lions) are our students, we do not really have that much of a problem. As said earlier, we are moving in a direction where students have increasing involvement in how and what they are taught. But if these *others* are the people outside mathematics and mathematics education, the policy makers and education managers, then we may end up *on the menu—*teaching mathematics in a way with which we do not agree.

## Footnotes

1 A social media stream is a feed of content that is collected from more than one platform and is most commonly displayed on a website.

2 *Educacio360 *is a national education initiative in Spain, with the aim of connecting all learning that takes place in the different teaching and non-teaching times and spaces, and of promoting personalised learning itineraries, inside and outside school. < https://www-educacio360-cat.translate.goog/?_x_tr_sl=ca&_x_tr_tl=en&_x_tr_hl=en&_x_tr_pto=sc#crida > .

3 *Remake Learning* is a network in Pennsylvania that ignites engaging, relevant, and equitable learning practices in support of young people navigating rapid social and technological change and involving different role players. < https://remakelearning.org/about/ > .
